# Environment as therapy: neuroatscience for intellectual disability and dementia

**DOI:** 10.18632/oncotarget.14602

**Published:** 2017-01-12

**Authors:** Gabriele Sansevero, Alessandro Sale

**Affiliations:** ^1^ Neuroscience Institute of CNR, National Research Council, Pisa, Italy

**Keywords:** neurodevelopmental disorders, dementia, Alzheimer's disease, environmental enrichment, plasticity, Neuroscience

Reversing conditions characterized by intellectual disability or dementia is one major challenge in basic and applied Neuroscience. This varied group of pathologies is highly heterogeneous and comprises, among others, neurodevelopmental disorders such as autism, Rett syndrome, and Down syndrome, and age-dependent neurodegenerative diseases such as Alzheimer’s disease (AD). Despite the heterogeneous etiology, recent research has underscored common cellular and molecular mechanisms underlying key neuropathological signs associated with these disorders, with emerging hub roles for GABAergic overinhibition, altered mGluR signaling, alterations in key neurotrophic factors, and dysfunctions in the metabolism of β-amyloid protein. The increased knowledge of molecular details has greatly expanded the attempts aimed at a pharmacological correction, with a growing number of studies reporting a marked phenotypic rescue in various transgenic mouse models of these disorders.

One paradigmatic case is that of Down syndrome (DS), a condition due to the presence of an extra copy of the human chromosome 21 and representing the most common genetic cause of intellectual disability. People with DS exhibit a complex phenotype characterized by a wide range of functional dysfunctions, encompassing attention and sensory deficits and moderate to severe mental retardation [1]. Moreover, subjects with DS invariantly show a precocious onset of AD, due to the triplication of the β-amyloid precursor protein gene, which is located on chromosome 21 [1]. This complex phenotype is faithfully recapitulated by the Ts65Dn mouse line, the most widely investigated animal model of DS, bearing a triplication for over half of the human chromosome 21 mouse gene orthologs. Recent attempts to ameliorate the phenotype of Ts65Dn mice have led to potentially interesting treatments as various as estrogen delivery, administration of GABA receptor or NMDA receptor antagonists, fluoxetine, lithium, acetylcholinesterase inhibitors, beta-adrenergic receptor agonists, bumetanide, and Epigallocatechin-3-gallate [2]. Despite great efforts devoted to develop suitable therapeutic approaches, however, there is still no effective cure for DS, and some of the proposed pharmacological approaches, such as the direct modulation of GABA receptors, raise concerns on their potential for clinical application, given that in many occasions the proposed drugs have either been not approved by the FDA or have an undesired proconvulsant action.

**Figure 1:**
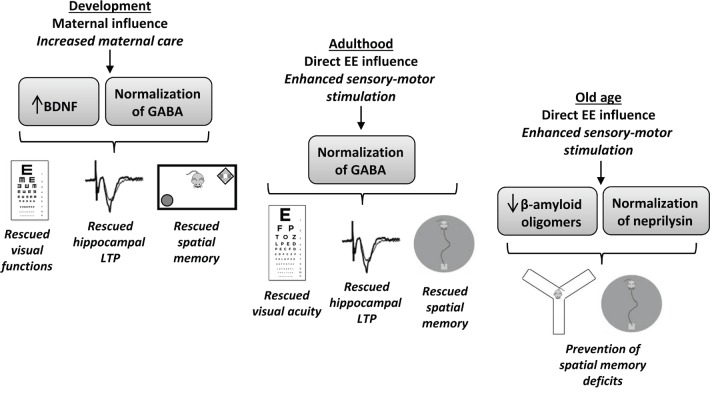
Impact of environmental enrichment in Ts65Dn mice

While awaiting for the good cocktail of pharma, mother Nature can go to Nurture’s school to get some help, or hints, or a combination of the two. In the last decades environmental enrichment (EE), a combination of sensory, motor and cognitive stimulation, has emerged as a valuable tool capable to stimulate cerebral plasticity both during the development and in the adulthood, with a totally non-invasive approach that renders this paradigm particularly interesting in terms of application to humans. EE exerts powerful effects on neural connectivity, enhancing hippocampal synaptic plasticity and learning and memory abilities, and positively interacting with various factors involved in neural plasticity, including NMDA receptors, inhibitory circuitry, intracellular kinases, neuromodulators, hippocampal neurogenesis, transcriptional and epigenetic factors, and neurotrophins [3]. Focusing on DS, we recently demonstrated that exposure to EE in adult Ts65Dn mice normalizes the intracerebral inhibitory-excitatory balance and restores hippocampal synaptic plasticity, learning abilities and visual functions [4] (see Figure 1). Similar effects were also reported in younger animals reared under EE conditions from birth, which received, compared to standard-reared age-matched controls, enhanced levels of maternal care, one of the most important sources of sensory experience during the early postnatal period [5]. The sooner, the better. Accordingly, early interventions based on EE have been also shown to correct brain development in other developmental disorders, such as Fragile X syndrome, Rett syndrome, and visual system deficits [3].

Strikingly, the beneficial effects of EE are not restricted to early life stages. Long-term exposure to EE prevents, in the hippocampus of aged Ts65Dn mice, the age-dependent increase of low molecular weight Aβ oligomer isoforms, the most dangerous molecular species triggering synapse failure and memory impairment in AD-like dementia [6]. Aged enriched Ts65Dn mice, compared to controls reared in standard conditions, show learning and memory performances that do not differ from those achieved by wild-type, euploid, controls [6] (see Figure 1). Analogously, EE has been also found to either prevent or reverse many neuropathological and behavioral signs of AD in several transgenic mouse models of this pathology [3].

Normalization of development, reversion of adult dysfunctions, prevention of AD-like neuropathology in elderly subjects: these remarkable effects should not be held as a miracle, as they rely on the EE ability to impact at several molecular substrate levels in the brain, including stimulation of maturational processes by enhancing the activation of growth factors, reopening of plasticity windows through reduced intracortical inhibition and promoting plastic structural and functional changes through neurotrophin increments, which could in turn increase the expression of genes specifically involved in brain plasticity.

These studies have profound consequences for humans. The development of intervention protocols aimed at ensuring proper levels of environmental stimulation and at maintaining a healthy and active lifestyle has been effectively encouraged by basic research on EE. A recent study in humans showed that an early multisensory intervention, based on infant body massage and directly inspired by research on maternal stimulation in rodents, promotes a significantly higher visual acuity in DS children at 6 months of age, leading to an accelerated development which persists up to at least 12 months [7]. Strong correlative and epidemiological evidence shows that a higher level and variety of mental and physical activity is associated with lower cognitive decline and reduced risk for dementia [3]. Most informatively, multidomain interventions based on enhanced exercise and cognitive training have been proved highly effective in improving cognitive performances not only in aged, healthy people, but even in Mild Cognitive Impairment subjects at high risk to develop dementia [8].

Future treatments in this field should combine the administration of new pharmacological therapeutics with an endogenous pharmacotherapy based on the intrinsic capability of environmental stimulation to enhance the spontaneous reparative potential held by the brain.
